# Enabling multi-level relevance feedback on PubMed by integrating rank learning into DBMS

**DOI:** 10.1186/1471-2105-11-S2-S6

**Published:** 2010-04-16

**Authors:** Hwanjo Yu, Taehoon Kim, Jinoh Oh, Ilhwan Ko, Sungchul Kim, Wook-Shin Han

**Affiliations:** 1 CSE Department, POSTECH, Pohang, South Korea; 2 CE Department, Kyungpook National University, Daegu, South Korea

## Abstract

**Background:**

Finding relevant articles from PubMed is challenging because it is hard to express the user's specific intention in the given query interface, and a keyword query typically retrieves a large number of results. Researchers have applied machine learning techniques to find relevant articles by ranking the articles according to the learned relevance function. However, the process of learning and ranking is usually done offline without integrated with the keyword queries, and the users have to provide a large amount of training documents to get a reasonable learning accuracy. This paper proposes a novel multi-level relevance feedback system for PubMed, called RefMed, which supports both ad-hoc keyword queries and a multi-level relevance feedback in real time on PubMed.

**Results:**

RefMed supports a *multi-level relevance feedback* by using the RankSVM as the learning method, and thus it achieves higher accuracy with less feedback. RefMed "tightly" integrates the RankSVM into RDBMS to support both keyword queries and the multi-level relevance feedback in real time; the tight coupling of the RankSVM and DBMS substantially improves the processing time. An efficient parameter selection method for the RankSVM is also proposed, which tunes the RankSVM parameter without performing validation. Thereby, RefMed achieves a high learning accuracy in real time without performing a validation process. RefMed is accessible at http://dm.postech.ac.kr/refmed.

**Conclusions:**

RefMed is the first multi-level relevance feedback system for PubMed, which achieves a high accuracy with less feedback. It effectively learns an accurate relevance function from the user’s feedback and efficiently processes the function to return relevant articles in real time.

## Background

PubMed is one of the most important information sources for biomedical researchers. It supports an efficient processing of keyword and constraint queries. However, finding relevant articles from PubMed is still challenging because it is hard to express the user’s specific intention in the given query interface, and a keyword query typically retrieves a large number of results. For example, the keyword “breast cancer” returns more than two hundred thousand articles. Adding a few more constraints could narrow down the search results but is still likely to return more results that the user can easily handle. The user can sort the results according to publication date, author’s first or last name, or journal name, but sorting them by some notion of relevance is hard.

To improve the search quality on PubMed, researchers have studied querying methodologies for PubMed, such as how to use controlled vocabulary, MeSH terms, or background knowledge to formulate proper PubMed queries [1,2]. Re-organizing the search results using ontologies or clustering techniques has been explored to provide better presentation of the results to the users [3-5]. Text mining researchers have also tried to compute the global importance of articles using the citation information and have applied it to rank the results as done in Google [4,6,7]. However, users’ specific intentions are typically widely varied even with the same keyword query. For example, with a query “breast cancer”, one user may be interested in finding genetic-study related papers while another user may want to find the latest cancer treatments. Thus ranking according to the global importance often does not meet the users’ specific information needs.

Researchers have also applied machine learning techniques to find relevant articles by ranking the articles according to the learned relevance function [8,9]. However, the process of learning and ranking is usually done offline without being integrated with the PubMed’s keyword queries, and the users have to provide a large amount of training articles to get a reasonable learning accuracy.

Finally, relevance feedback, a well established technique in IR to improve retrieval performance [10,11], has been applied on PubMed (e.g., MiSearch, a recent relevance feedback system for PubMed [12]). However, existing relevance feedback systems use classification methods and thus are limited to two level relevance judgements (relevant or not).

This paper proposes a novel multi-level relevance feedback system for PubMed, called RefMed, which supports both ad-hoc keyword queries and a multi-level relevance feedback in real time on PubMed.

**Figure 1:**
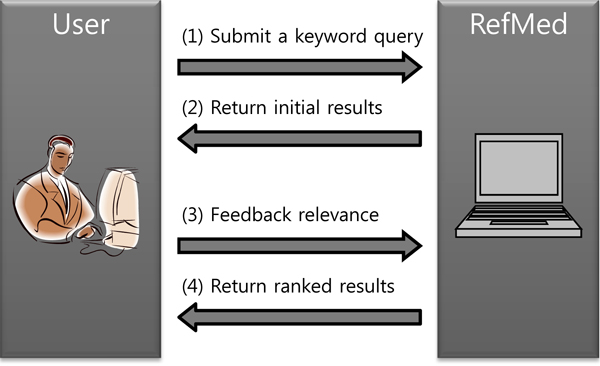
Search process in RefMed

Figure 1 shows the search process in RefMed. RefMed first accepts a keyword query (Step 1) and returns initial results (Step 2) as done in PubMed. While browsing the resulting documents, the user makes relevance judgments on some of them (Step 3). The number of relevance levels is set to three as default but can be adjusted depending on the user’s preference. Once the user “pushes the feedback,” the system induces a relevance function from the feedback using the RankSVM [13] and returns top-k results ranked according to the function (Step 4). The user can repeat this process until she receives satisfying results. This process of learning and ranking is done in real time.

To the best of our knowledge, RefMed is the first “multi-level” relevance feedback system for PubMed. The new technical contributions of RefMed are as follows.

• RefMed supports the multi-level relevance feedback by using the RankSVM as the learning method, and thus it achieves higher accuracy with less feedback. Traditional relevance feedback systems use classification methods for learning (e.g., SVM, Bayesian learning) and thus are limited to two levels of relevance judgments (i.e., relevant or not). RankSVM is one of the most actively researched algorithms for learning ranking functions in the machine learning community and is regarded as the most accurate methodology when the size of training data is relatively small [13]. In a real time relevance feedback system such as RefMed, the amount of user feedback, i.e., training data, is typically small. Thus, we adopted the RankSVM as the learning method.

• RefMed “tightly” integrates the RankSVM into a relational database management system (RDBMS) to support keyword queries and relevance feedback in the same framework and to minimize the response time. Specifically, we develop and integrate new SQL expressions for learning and predicting ranking into DBMS. The tight coupling of RankSVM and DBMS improves the processing time substantially by running the RankSVM directly on the data tables instead of files. The new SQL expressions also facilitates the application development process by running the rank learning and predicting operations within SQL.

• An efficient parameter selection method for RankSVM is proposed, which tune the parameter without performing validation. Validation is a necessary process in learning with RankSVM in order to tune the soft margin parameter *C*. However, it is not feasible to perform the validation in RefMed, as no validation set is given during the search process. By the parameter selection method, RefMed estimates the best parameter to achieve a high learning accuracy without performing validation.

Methods section overviews the RankSVM and presents the integration of RankSVM within SQL and our parameter selection method. Result section demonstrates RefMed, and reports experiment results. We report (1) the learning accuracy of RankSVM against Rocchio with different amounts of feedback and relevance levels, (2) the query processing time of the tight coupling against a loose coupling, and (3) the accuracy of our parameter selection method against the cross validation and other parameter selection methods.

## Methods

### Preprocessing PubMed

Each PubMed article is structured with multiple attributes such as title, abstract, publication date, journal name, author names, MeSH terms, etc. We extract features from the title and abstract of each article because a user tends to make a relevance judgment based on them. Specifically, each article vector  is represented by a set of TFIDF scores of the words extracted from the title and abstract such that:

Stopwords and word stemming are processed before extracting the features.

### RankSVM

Let "*A* is preferred to *B* " be specified as "". A training set for RankSVM is denoted as  where *y_i_* is the ranking of , that is, *y_i_ < y_j_* if . Given a training set *R*, RankSVM computes a rank scoring function *F* such that  for any . For now, assume *F* is a *linear* ranking function such that:

 (1)

Then, the goal is to learn *F* which is concordant with the ordering *R* and also generalize well beyond *R*. That is to find the weight vector  such that  for most data pairs . RankSVM finds such weight vector by solving the following optimization problem [14]

	minimize:	 (2)

	subject to:	 (3)

 (4)

By the constraint (3) and by minimizing the upper bound  in (2), the above optimization problem satisfies orderings on the training set *R* with minimal error. By minimizing  or by maximizing the margin (), it tries to maximize the generalization of the ranking function. *C* is the soft margin parameter that controls the trade-off between the margin size and training error. (Refer to Conclusion section of [15] for more detailed explanation about formulating the optimization problem of RankSVM.)

The primal problem of RankSVM can be transformed to the following dual problem using the Lagrange multipliers.

	maximize:	 (5)

	subject to:	 (6)

Once transformed to the dual, the kernel trick can be applied to support nonlinear ranking function. *K* (·) is a kernel function where *K* (*a,b*) = *a·b* in the linear kernel or  in the RBF kernel. The RBF kernel contains an additional parameter *g* that needs to be tuned. (Refer to Methods section of [15] for more detailed explanation of the kernel trick.)

α*_ij_* is a coefficient for a pairwise difference vectors . Once α is computed,  can be written in terms of the pairwise difference vectors and their coefficients such that:

 (7)

The pairwise difference vectors whose coefficients α > 0 are support vectors. The ranking function *F* on a new vector  can be computed using the kernel function replacing the dot product as follows:

 (8)

The function *F* becomes a linear function w.r.t. the features when *K* is the linear kernel, or *F* is a nonlinear function when *K* is a nonlinear kernel such as the RBF kernel. RefMed applies the linear kernel since the linear kernel is known to perform well for high dimemsional data such as documents [16].

### Integration of RankSVM within SQL

RefMed tightly integrates the RankSVM into the MySQL DBMS in order to minimize the response time. The tight integration of RankSVM enables the learning and processing of ranking on the SQL data tables directly without additional disk accesses for generating intermediate files. By integrating RankSVM within MySQL, we can also use the DB facilities such as indexes and optimizers for managing and accessing the data.

**Figure 2:**
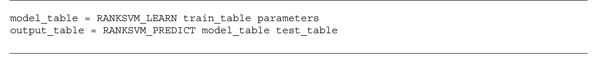
SQL commands for RankSVM learning and predicting

RankSVM runs two operations — learning and predicting, thus we developed two new SQL commands for RankSVM as Figure 2 shows, and embedded them into SQL. RANKSVM_LEARN has *train_table* and *parameters* as inputs and *model_table* as the output. RANKSVM_PREDICT has *model_table* and *test_table* as inputs and *output_table* as the output.

**Figure 3:**
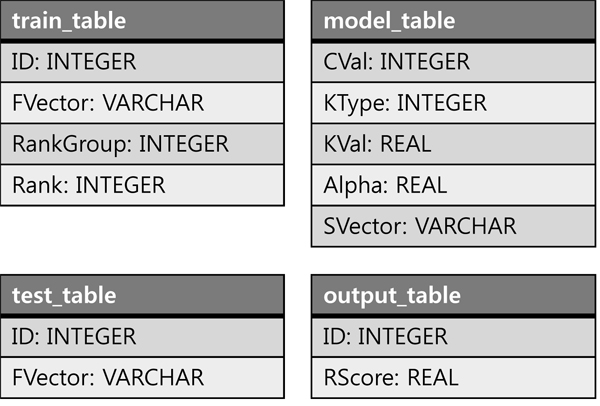
Table schema for *train_table*, *model_table*, *test_table*, and *output_table*

Figure 3 shows the schema of *train_table*, *model_table*, *test_table*, and *output_table*. The *train_table* contains four attributes — the instance id (ID), feature vector describing the instance (FVector), and the ranking label of the instance (RankGroup and Rank). Note that both RankGroup and Rank are needed to specify the ranking label of instances in a set of relative orderings. The *parameters* consists of soft margin parameter (CVal), kernel type (KType), and kernel parameter (KVal).

The *model_table* is constructed after running RANKSVM_LEARN, which contains the model information and will be used as an input of the RANKSVM_PREDICT command. The model information includes the parameters (i.e., CVal, KType, and KVal) and a set of support vectors and the coefficients (i.e.  and α*_ij_* in Eq.(7)), The *test_table* contains attributes ID and FVector, and The *output_table* contains attributes ID and RScore (the ranking score).

**Figure 4:**
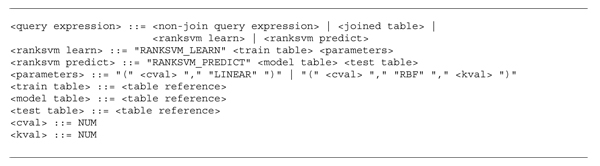
SQL BNFs for RankSVM learning and predicting

Figure 4 shows the corresponding SQL BNFs for RANKSVM_LEARN and RANKSVM_PREDICT. We currently support the linear and RBF kernels, that are, the two most popularly used kernels. Note that, the learning and predicting commands are defined as parts of the <query expression> of SQL, and thus they can be used as a subquery of another SQL query. The train table, model table, and the test table are defined as the <table reference> of SQL, and thus a subquery can be placed within the commands.

**Figure 5:**
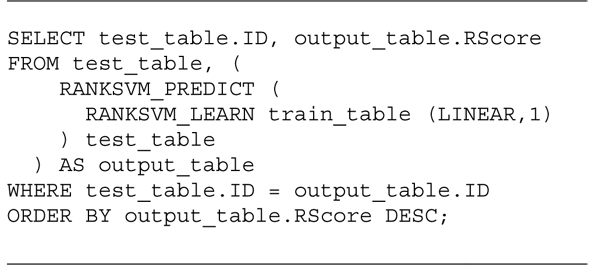
An SQL query example using RANKSVM_LEARN and RANKSVM_PREDICT

Figure 5 shows an example of SQL query using the rank commands to rank the data in *test_table* according to the function learned from *train_table*. The RANKSVM_LEARN statement is first processed and returns a model table that becomes the first argument of the RANKSVM_PREDICT. The output of the RANKSVM_PREDICT is renamed as the *output_table*, which is joined with the *test_table* to generate the results ranked according to the *RScore* in an descending order.

### Parameter selection

RankSVM has the soft margin parameter *C* (in Eq.(2)) that controls the tradeoff between the margin size and training error. The parameter typically needs to be tuned by a validation process, but it is infeasible to perform a validation process in a real-time relevance feedback system such as RefMed, as no validation set is provided. We develop a parameter selection method for RankSVM that tunes the soft margin parameter without running a validation process as follows.

From Eq.(8) and Eq.(6),

Since *K* (·) > 0,

Thus,

 (9)

Assume the training set is a set of multi-level relevance levels (e.g., {1:"not relevant", 2:"partially relevant", 3:"highly relevant"}) where F (a) > F (b) for . Then, we can estimate the lower bound of *C* by computing Eq.(9) using the training set. In fact, when  is a bounded support vector whose α = *C*, the inequality in Eq.(9) becomes the equality. The summation in the denominator is over all records in the training set. The resulting set of *C* values is sorted in descending order, and the 90^*th*^ percentile value is selected. Thus, the model is given sufficient capacity to achieve good but not perfect performance for the training data.

Our experiments (in "Evaluation of Parameter Selection" Section) show that our parameter selection method generates significantly higher accuracy than the default parameters provided in the SVM light [13] and LibSVM [17], and its accuracy is very close to that of the cross validation.

## Results

### RefMed demonstration

**Figure 6:**
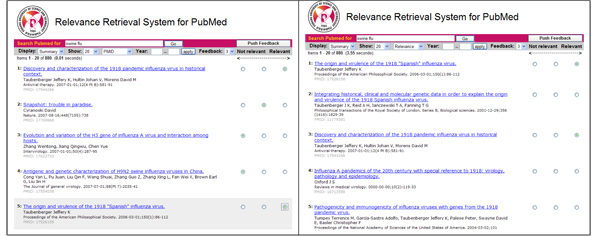
Feedback relevance (left) and ranked results (right) in RefMed

Figure 6 (left) shows the screen shot of the user's feedback on the results of query "swine flu" in RefMed. Just as PubMed, RefMed first shows 20 articles of 880 results sorted by PMID (PubMed ID) in the first page. The user marked the first and fifth article as *Relevant* and the third and fourth articles as *Not relevant*. Once the user presses the "Push Feedback" button, RefMed (1) learns a relevance function, (2) sorts the 880 articles according to the function, (3) and returns top 20 articles. Figure 6 (right) shows the screen shot of the top 20 articles. Note that the first and third articles are those that the user marked as *Relevant* previously. The user can keep judging the relevance on the other articles until she receives satisfying results.

### Accuracy evaluation

This section evaluates the effectiveness of the multi-level relevance feedback over binary relevance feedback. The effectiveness is measured based on NDCG and Kendall's τ, that are, the two popularly used measures for evaluating ranking accuracy. NDCG is popularly used for IR applications where ranking on top results is more important than that on bottom results [18-21], and Kendall's τ is favorably used to measure the overall accuracy based on the number of correctly ranked pairs [22-24]. There are other measures for evaluating the ranking accuracy such as AUC (Area Under the Curve) and MAP (Mean Average Precision). They are used when there are two levels of relevance. Descriptions of Kendall's τ and NDCG follow.

#### Kendall's τ

 Let R^*^ be the optimal ranking of data in which the data is ordered perfectly according to the user's preference. A ranking function *F* is evaluated by how closely its ordering *R^F^* approximates R^*^. Kendall's τ has been a widely used measure for similarity between two orderings R^*^ and *R^F^*[22,25]. For two strict orderings *R^a^* and *R^b^*, Kendall's τ is defined based on the number *P* of concordant pairs and the number *Q* of discordant pairs. If R^*^ and *R^F^* agree in how they order a pair ,  and , the pair is concordant, otherwise, it is discordant. For ordering R^*^ and *R^F^* on a dataset *D*, we define the similarity function τ as the following, which is equivalent to that defined in [25]:

 (10)

To illustrate, suppose R^*^ and *R^F^* order five vectors  as follow:

 (11)

 (12)

In this example, τ (R^*^, *R^F^*) is computed as 0.7, as the number of discordant pairs is 3, i.e.,  while all remaining 7 pairs are concordant.

#### Normalized Discount Cumulative Gain (NDCG)

While the Kendall's τ computes the overall accuracy of a ranking function, NDCG focuses on the accuracy on the top results rather than on the bottom results. For example, when users rated movies from 1 (meaning 'terrible') to 5 (meaning 'excellent'), the learning machine learned a ranking function from the training data and returned top *n* results. Then, NDCG score is computed as follows.

 (13)

Where *j* is the position from the top, R(*j*) is the rating of the *j* 's movie. *Z_n_* is a normalization factor to guarantee that the NDCG score of a perfect ranking is equals to 1. As *j* increases or as the returned movie becomes farther from the top, its impact on the NDCG score decreases logarithmically.

#### Data sets

We used synthetic and OHSUMED data sets. The synthetic data consists of 150 data instances of 50 features and each feature value is a random number between zero and one. A linear function is then created by generating a random weight vector  in . Then, the training and testing set are created using the function *F*, and the accuracy is measured by comparing the *F' * and on testing set where *F' * is learned from the training set.

The OHSUMED data set is a subset of the PubMed articles and consists of 348,566 documents and 106 queries [26]. In total, there are 16,140 query-document pairs on which relevance judgments are made. The relevance judgments are either 'd' (definitely relevant), 'p' (partially relevant), or 'n' (not relevant). The data has been used in many experiments in IR [21]. In the same way we preprocessed the PubMed data, we preprocessed the OHSUMED documents and extracted features by running stopwords, word stemming, and computing TFIDF. A feature is a TFIDF value for each word.

#### Results

**Figure 7:**
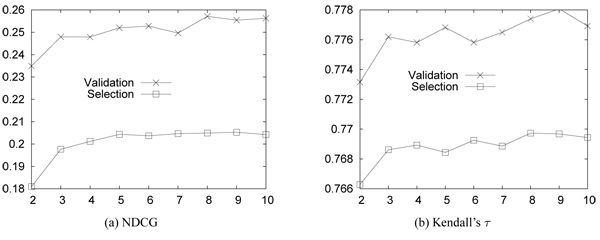
**Accuracy on synthetic data.** X-axis: number of relevance levels; Y-axis: accuracy (NDCG and Kendall’s τ ). “Validation”: 3-fold cross validation; “Rank-Selection”: our parameter selection method

Figure 7 shows the accuracy (i.e., NDCG and Kendall's τ) of RankSVM with varying number of relevance levels on the synthetic data. "Validation" and "Rank-Selection" are the results of 3-fold cross validation and our parameter selection method. The results are averaged over 30 runs. *As the relevance level increases, the accuracy increases substantially in the beginning, but the increments become insubstantial.*

**Figure 8:**
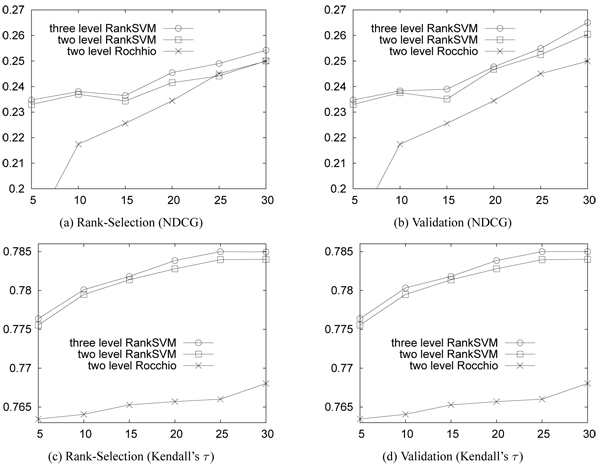
**Accuracy on OHSUMED data.** X-axis: number of training documents; Y-axis: accuracy (NDCG and Kendall's τ). "Validation": 3-fold cross validation; "Rank-Selection": our parameter selection method

Figure 8 shows the accuracy (i.e., NDCG and Kendall's τ) with varying size of training documents on the OHSUMED data. We used the first 30 queries for this experiment. For each query, we varied the number of training documents and compared the accuracy of the three-level RankSVM, two-level RankSVM, and two-level Rocchio. Note that Rocchio is a classification method learning from only the two-levels of data while RankSVM can learn from various number of levels of data [12]. Since the OHSUMED data set contains three levels of relevance, we created the data of two levels by merging "highly relevant" and "partially relevant" into "relevant". The results are averaged over 30 runs on the 30 queries (averaged over 900 results). Figure 8(a) and 8(c) show the accuracy when the parameter is tuned using our parameter selection method, and 8(b) and 8(d) show the accuracy when the parameter is tuned using the 3-fold cross validation. The accuracy increases as the training size increases, and *the training data with three relevance levels always generates higher accuracy than that with two levels.The RankSVMs significantly outperform the Rocchio especially when the size of training data is small*.

### Efficiency evaluation

We compare the efficiency of the tight coupling against the loose coupling. In the loose coupling, DBMS exports the data in the tables to files, RankSVM trains and predicts on the files, the prediction results are imported to the DBMS tables, and the DBMS processes the rest of the query. We ran the experiments using the MySQL v5.0.5 on a linux machine with two Intel Quadcore CPUs, 48GB memory, and 4.5TB HDD.

**Figure 9:**
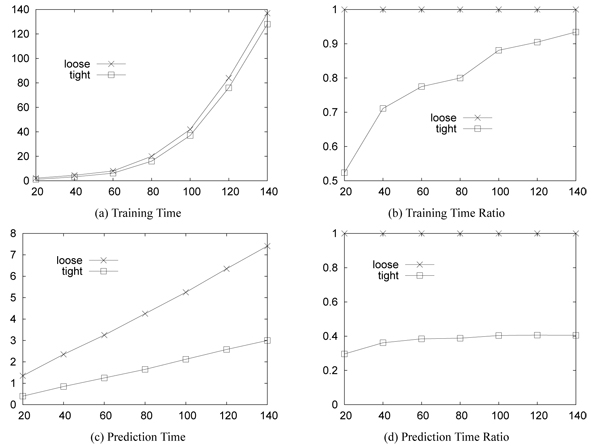
**Efficiency comparison between loose and tight coupling.** X-axis: size of data; Y-axis: time in msec.

Figure 9 compares the training and prediction times of the loose and tight coupling as the data size increases. Figure 9(a) shows the actual training time of the methods and 9(b) shows the training time ratio of the methods where the training time of the loose coupling is one. While the training time difference between them is significant when the data size is small, it becomes trivial as the data size increases. It is because the training time complexity of RankSVM is polynomial to the data size. However, as Figure 9(c) and 9(d) show, the prediction time is reduced 60% overall by the tight coupling regardless of the data size. The prediction time of RankSVM increases linearly as the data size increases. The results are averaged over 30 runs on the OHSUMED data.

### Evaluation of parameter selection

We compare five different parameter selection methods — (1) Rank-Selection: our parameter selection method, (2) C-Selection: the method proposed in [27], (3) SVM-Light: the svm light default parameter (), (4) LIBSVM: the libsvm default parameter (= 1), and (5) CV: 3-fold cross validation.

We grouped six queries of documents — qid 1-6, qid 7-12, and qid 13-18, and evaluated the parameter selection methods on the grouped data by measuring the RankSVM accuracy with the parameter selected by each method. Table 1 shows the accuracies of the five different parameter selection methods. Note that only the cross validation uses validation sets for parameter tuning, thus it shows the highest accuracy. Among the other four methods that do not use validation sets, our Rank-Selection method achieves the highest accuracy.

**Table 1:** Accuracy of RankSVM with five different parameter selection methods

NDCG@10					
	**SVM-Light**	**LIBSVM**	**C-Selection**	**Rank-Selection**	**CV**

qid 1-6	0.472788	0.471753	0.493179	**0.654761**	0.66833
qid 7-12	0.386431	0.390575	0.377642	**0.454554**	0.45718
qid 13-18	0.320041	0.320191	0.31299	**0.35037**	0.37794

Kendall's *tau*					

qid 1-6	0.787682	0.786749	0.789671	**0.817379**	0.818743
qid 7-12	0.786614	0.786388	0.788733	**0.803645**	0.804825
qid 13-18	0.780289	0.779738	0.781045	**0.782841**	0.78917

## Conclusions

This paper proposes RefMed, a novel multi-level relevance feedback system for PubMed. RefMed supports the multi-level relevance retrieval by using the RankSVM as the learning method. RefMed tightly integrates the RankSVM into RDBMS to support both keyword queries and relevance feedback in real time. A novel parameter selection method for the RankSVM is also proposed, which tune the soft margin parameter without performing a validation process. By the tight coupling of RankSVM within DBMS and the parameter selection method, RefMed achieves a high relevance accuracy with less feedback.

## Competing interests

Authors declare that they have no competing interests.

## Authors' contributions

HY, TK, JO, IK, and SK designed and implemented the RefMed. WH designed the SQL integration for RankSVM.
